# THz Water Transmittance and Leaf Surface Area: An Effective Nondestructive Method for Determining Leaf Water Content

**DOI:** 10.3390/s19224838

**Published:** 2019-11-06

**Authors:** Mario Pagano, Lorenzo Baldacci, Andrea Ottomaniello, Giovanbattista de Dato, Francesco Chianucci, Luca Masini, Giorgio Carelli, Alessandra Toncelli, Paolo Storchi, Alessandro Tredicucci, Piermaria Corona

**Affiliations:** 1 CREA—Research Centre for Plant Protection and Certification, Via di Lanciola 12/A, 50125 Firenze, Italy; 2 CREA—Research Centre for Viticulture and Enology, Viale Santa Margherita 80, 52100 Arezzo, Italy; paolo.storchi@crea.gov.it; 3 NEST, CNR—Istituto Nanoscienze and Scuola Normale Superiore, Piazza San Silvestro 12, 56124 Pisa, Italy; arriva.bazza@gmail.com (L.B.); andrea.ottomaniello@df.unipi.it (A.O.); luca.masini@sns.it (L.M.); alessandro.tredicucci@unipi.it (A.T.); 4 Dipartimento di Fisica “E. Fermi”, Università di Pisa, Largo Bruno Pontecorvo 3, 56127 Pisa, Italy; giorgio.carelli@unipi.it (G.C.); alessandra.toncelli@unipi.it (A.T.); 5 CREA—Research Centre for Forestry and Wood, Viale Santa Margherita 80, 52100 Arezzo, Italy; giovanbattista.dedato@crea.gov.it (G.d.D.); francesco.chianucci@crea.gov.it (F.C.); piermaria.corona@crea.gov.it (P.C.)

**Keywords:** leaves, plants, terahertz quantum cascade laser, water content, drought stress

## Abstract

Water availability is a major limiting factor in plant productivity and plays a key role in plant species distribution over a given area. New technologies, such as terahertz quantum cascade lasers (THz-QCLs) have proven to be non-invasive, effective, and accurate tools for measuring and monitoring leaf water content. This study explores the feasibility of using an advanced THz-QCL device for measuring the absolute leaf water content in *Corylus avellana* L., *Laurus nobilis* L., *Ostrya carpinifolia* Scop., *Quercus ilex* L., *Quercus suber* L., and *Vitis vinifera* L. (cv. Sangiovese). A recently proposed, simple spectroscopic technique was used, consisting in determining the transmission of the THz light beam through the leaf combined with a photographic measurement of the leaf area. A significant correlation was found between the product of the leaf optical depth (*τ*) and the leaf surface area (L_A_) with the leaf water mass (M_w_) for all the studied species (Pearson’s *r* test, *p* ≤ 0.05). In all cases, the best fit regression line, in the graphs of *τ*L_A_ as a function of M_w_, displayed R^2^ values always greater than 0.85. The method proposed can be combined with water stress indices of plants in order to gain a better understanding of the leaf water management processes or to indirectly monitor the kinetics of leaf invasion by pathogenic bacteria, possibly leading to the development of specific models to study and fight them.

## 1. Introduction

Plants are continuously exposed to multiple stresses such as drought, salinity, extreme temperatures, nutrient deficiencies, mineral toxicities, and attacks from pathogens (e.g., fungi), of which drought is the most important factor limiting growth and plant production [1]. Plants can fight a reduction of plant-available water and preserve osmotic potential by reducing stomata conductance [2]. As a matter of fact, leaves are the main organs regulating plant water balance [3] and physiological activity [4]. The balance between leaf vein density, which is linked to hydraulic conductance and water supply [5,6,7,8], and stomatal density and size regulates maximum conductance and transpiration [9]. Recent studies have highlighted the key role the leaf venation network plays in photosynthesis [10,11] and leaf temperature regulation [12]. Leaf hydraulic architecture plays a key role in sap flow [13] as the water content in vegetative tissues is an extremely important parameter for photosynthetic performance [2]. 

Recent research has shown indices that can be used for characterizing the leaf water content, such as the full moisture content (FMC) [14] and the relative water content (RWC) [15,16]. Leaf water potential, which is defined as the measure of free energy per unit volume, is a useful variable for estimating leaf water status [17]. Leaf water potential and the previously described indices generally require destructive sampling of the leaf, although leaf water potential can also be estimated using nondestructive leaf psychrometry. However, this nondestructive technique shows data inconsistencies with standard chamber pressure measurements under field conditions [18]; in fact, it generally yields higher values. Another widely used technique is the gravimetric analysis, defined as the ratio of leaf water content to leaf dry or fresh mass [19], which is widely used for measuring leaf water content, but it is time-consuming and destructive. 

Using the method proposed in this study, it is possible to measure leaf water content in a fast and nondestructive way. Although this parameter is not a metric indicating plant water status, it is an important parameter that can be combined with other information such as near-infrared reflectance or sap flow data in order to gain better insight into the water management processes of the plant. Moreover, thanks to it being inherently nondestructive, this measurement can be carried out in real time to monitor both short- and medium-term variations of this parameter over time on the same leaf or different leaves and/or different plants, thus broadening the scope of new genetic or physiological experiments. It may also be useful for investigating and predicting the transpiration rate of novel genetic lines producing beneficial results on plant response to abiotic stimuli [20]. It is also possible to combine the leaf water content data with the other parameters listed above, in order to obtain indirect information on the stem water content, which provides a buffering capacity for maintaining plant hydraulic functionality. Our method could be used for carrying out experiments on anisohydric and isohydric plant behavior. For example, anisohydric plants generally exhibit less stomatal sensitivity to evaporative demand and soil moisture, inducing intensive fluctuations in leaf water potential [21]. Experiments on isohydric and anisohydric plant behavior can be carried out using the abscisic acid hormone combined with our technological approach for monitoring the transpiration process under abiotic stress conditions. This method could also be used for studying real time leaf water loss due to transpiration under various environmental conditions (e.g., light quality) [22]. It is also possible to use our method for studying other aspects of plant kinetics such as the invasion of leaves by living organisms. In fact, leaf blade water content data could prove useful for evaluating the diffusion of pathogens [23]. 

Much research attention has been directed towards terahertz [24] imaging and sensing techniques [25], such as time-domain spectroscopy [26], confocal microscopy [27] and terahertz quantum cascade lasers (THz-QCL) [28]. These new technologies can help us to obtain non-invasive, fast, and reliable assessments of leaf hydration status. Terahertz radiation is particularly promising thanks to the high water absorption coefficient and the relatively low absorption coefficient of the leaf dry-matter content [29,30]. Its particularly long wavelength makes this type of radiation relatively insensitive to scattering from leaf dry mass; consequently, THz imaging has proven to be an effective tool for non-invasive monitoring of plant water content [8,31,32]. Moreover, THz imaging can be used for mapping water density in single leaves [27,33], for carrying out time-lapse studies on leaf and trunk hydration dynamics [34,35], and for monitoring plant drought stress response [36]. Other spectral methods such as infrared spectroscopy [14,37] and hyperspectral spectroscopy [38] are currently used to obtain a large amount of useful information (i.e., water data) thanks to their high levels of automation and engineering. Their main drawback is that they require particular wavelengths or combinations of indices in order to obtain a strong correlation with actual water data. 

In this study, we tested the effectiveness of using a terahertz quantum cascade laser (THz-QCL) for measuring the optical transmission of leaves and the leaf surface area (L_A_) as a nondestructive method for estimating the absolute leaf water content in plant species with different leaf traits. This method has previously been successfully used to measure leaf water content in a single variety of *Vitis vinifera* L. [28]. However, using the method for other plant species may prove problematic, especially for waxy leaves. In addition to reducing the signal-to-noise ratio of the THz signal, a non-negligible THz absorption by the solid components of the leaf could affect the reliability of the water measurement through leaf-to-leaf and plant-to-plant variability and the ensuing fluctuations of residual absorption. Since THz absorption is a nondestructive technique, this issue would not affect the determination of relative metrics (i.e., the RWC) or time evolution measurements that can be performed sequentially on the same leaf. However, in some species, this might affect absolute calibration against established methods that rely on destructive approaches. In order to solve this issue, the same method was used to determine the leaf water content of six different plant species. The results were compared with direct destructive measurements obtained from gravimetric measurements in order to demonstrate that a strong correlation/calibration can indeed be obtained. 

## 2. Materials and Methods

### 2.1. Plant Material

The experiments were performed in three different periods of time: September 2017, October 2017, and February 2018. *Corylus avellana* L. (C_A_), *Ostrya carpinifolia* Scop. (O_C_), and *Vitis vinifera* L. (V_V_, cv. Sangiovese) were used in the first time period. *Quercus suber* L. (Q_S_) plants were used in the second time period, while *Laurus nobilis* L. (L_N_) and *Quercus ilex* L. (Q_I_) were used in the last time period. C_A_, O_C_, Q_S_, L_N_, and Q_I_ plants were cultivated at the Carabinieri Forestali nursery (Pieve Santo Stefano, Arezzo, Italy; N 43° 39′ 18.7” E 12° 03′ 27.5”) while V_V_ were cultivated at the New Plants nursery (Cenaia, Pisa, Italy; N 43° 36′ 23.7” E 10° 32′ 30,2”). Five plants were used for the first and third periods, while seven plants were used for the second. The first group of plants was exposed to the same environmental conditions (31.5 °C, 45.5% of relative humidity, approximately 336 μmol m^−2^ s^−1^ P.A.R.) inside a greenhouse of the physics department at the University of Pisa (Italy). The second group of plants was placed inside a plant growth chamber (21.0 °C, 50.0% of relative humidity, approximately 600 μmol m^−2^ s^−1^ P.A.R.) at the CREA Institute in Arezzo (Italy). The third group of plants was placed inside a greenhouse (12.3 °C, 48.2% of relative humidity and approximately 158 μmol m^−2^ s^−1^ P.A.R.) at the same institution. Photon flux measurements were conducted using an Em50 (Meter Group, Pullman, WA, USA) data logger equipped with a QSO-S PAR Photon Flux sensor (apogee Instruments Inc., Logan, UT, USA). The environmental conditions, temperature, and humidity were measured with a calibrated USB temperature and humidity data logger (model DS102, MISOL International E-commerce, Jiaxing City, China). The plants were healthy, and soils were watered to full-field capacity. 

### 2.2. Measurements of Water Content

The method proposed by Baldacci [28] was used for determining the leaf fresh mass which combines transmittance data from a terahertz quantum cascade laser (THz-QCL) with leaf surface data from images obtained using an RGB digital camera (EOS 1100D camera with EFS 18–55 mm 0.25 m/0.8 ft macro objective, Canon, Tokyo, Japan). 

**Figure 1:**
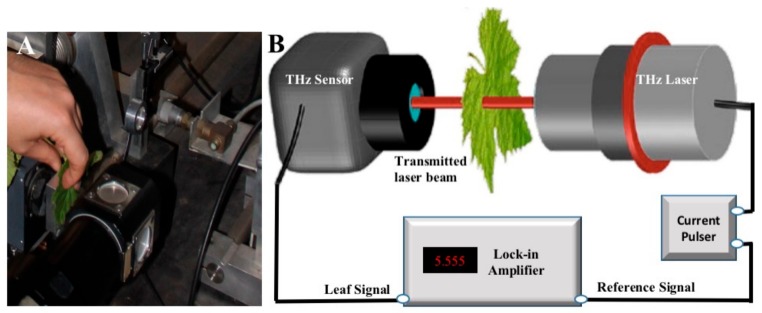
(**A**) THz leaf measurement method using a current pulser driving a THz cryo-cooled quantum cascade laser (QCL) to generate 2.55 THz laser radiation. (**B**) THz transmission measurement setup.

THz measurements were performed in the laboratory (~24 °C and 53% of relative humidity), using a simple transmission setup (Figure 1) with a 2.55 THz cryo-cooled QCL as the light source and a Golay cell detector (Hasen Consultant Ltd., Russia) to measure the THz signals. 

The signal was sent to a lock-in amplifier (SR830 Stanford Research Systems, Sunnyvale, CA USA) synchronized with the QCL light pulses. An aluminum screen with a reference hole along the path was inserted just before the detector in order to place the leaves in the same position, to collect the THz light waves from the same portion of space, and to reduce background scatter and noise. Only the adaxial leaf surfaces were exposed to the laser source. Optical measurements were taken on leaves randomly chosen from the main shoots of the potted plants. For each leaf, transmission data were taken from four different points on the adaxial leaf surface. Points were chosen as follows: One from the petiole sinus to the first bifurcation; one on the first order vase in the distal part; and two on the lateral lobes, left and right. Two points (one from the petiole sinus to the first bifurcation and one on the first order vase in the distal part) were used for the Q_S_ as the leaves were too small to be managed easily. The optical depth (*τ*) of each point on the leaf was calculated using the following Equation (1):

(1) $$\tau =\ln\left[\frac{I_{0}}{I_{Tr}}\right],$$

where *I*_0_ is the laser beam intensity incident on the leaf averaged over two measurements taken before and after the transmission measurements and *I_Tr_* is the laser beam intensity transmitted by the leaf averaged over four different measurements taken in different locations in the leaf, as described above. More details on this method can be found in Baldacci [28]. All of the THz measurements were performed with the leaf still attached to the plant. RGB images were obtained using an EOS 1100D camera (Canon, Tokyo, Japan) with EFS 18–55 mm 0.25 m/0.8 ft macro objective (Canon, Japan). After capturing the leaf shape on camera, the L_A_ was measured using ImageJ 1.50i software (National Institutes of Health, Bethesda, MD, USA). 

L_A_ processing consisted of the following steps: Open image, analyze, set scale, and select polygon (as a tool for outlining the leaf perimeter). For each plant, fully developed leaves were randomly chosen from the main shoot of the plants, ten of which were used for taking measurements of L_N_, V_V_, and O_C_. Eleven leaves were used for Q_I_ and C_A_, while seven leaves were used for O_S_ depending on the number of leaves on the main shoot. In order to investigate the relationship between the leaf optical properties and leaf water content (M_w_), the leaves were detached and weighted immediately after measuring the THz transmission, then dried at 105 °C and reweighted 24 h later. 

## 3. Results and Discussion

### 3.1. The Relation between τL_A_ and Leaf Water Mass 

**Figure 2:**
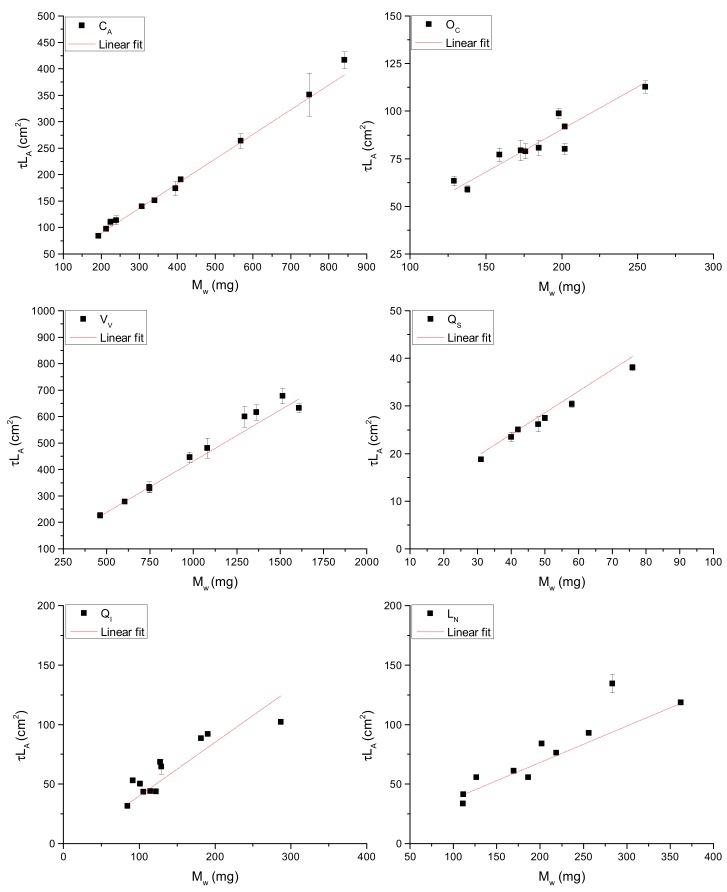
Relationships between the product of the leaf optical depth and the leaf surface area (*τ*L_A_) and the leaf water mass (M_w_) with respect to the investigated species.

A positive and significant correlation (Pearson’s r test, significant at *p* ≤ 0.05) was found between the product of the leaf optical depth (*τ*) and the leaf surface area (L_A_) with the leaf water content (M_w_) for all of the species analyzed as shown in Figure 2. It is important to note that data from different leaves of different plants are shown in each graph. The data of the slope (C_1_) of each relationship between *τ*L_A_ and M_w_ (*τ*L_A_ = C_1_M_w_ + C_0_) are highlighted as follows: C_A_ (0.47 ± 0.01 cm^2^ mg^−1^), O_C_ (0.45 ± 0.05 cm^2^ mg^−1^), V_V_ (0.39 ± 0.02 cm^2^ mg^−1^), Q_S_ (0.45 ± 0.04 cm^2^ mg^−1^), L_N_ (0.31 ± 0.03 cm^2^ mg^−1^), Q_I_ (0.45 ± 0.06 cm^2^ mg^−1^). Table 1 shows leaf water content (M_w_), leaf area (L_A_), leaf dry mass (LDM), and specific leaf area (SLA), that is, the ratio of leaf area to leaf dry mass of each species. Table 2 shows the optical depth and the adjusted R^2^ values of the linear regression model of each species. As shown in Table 2, in all cases, the adjusted R^2^ values of the correlations between *τ*L_A_ and M_w_ are always greater than 0.85. This result is in line with previous research findings in V_V_ [28] and shows that the method can be used for all the species under study.

### 3.2. The Effectiveness of the Approach Used for Measuring the Leaf Water Content

**Figure 3:**
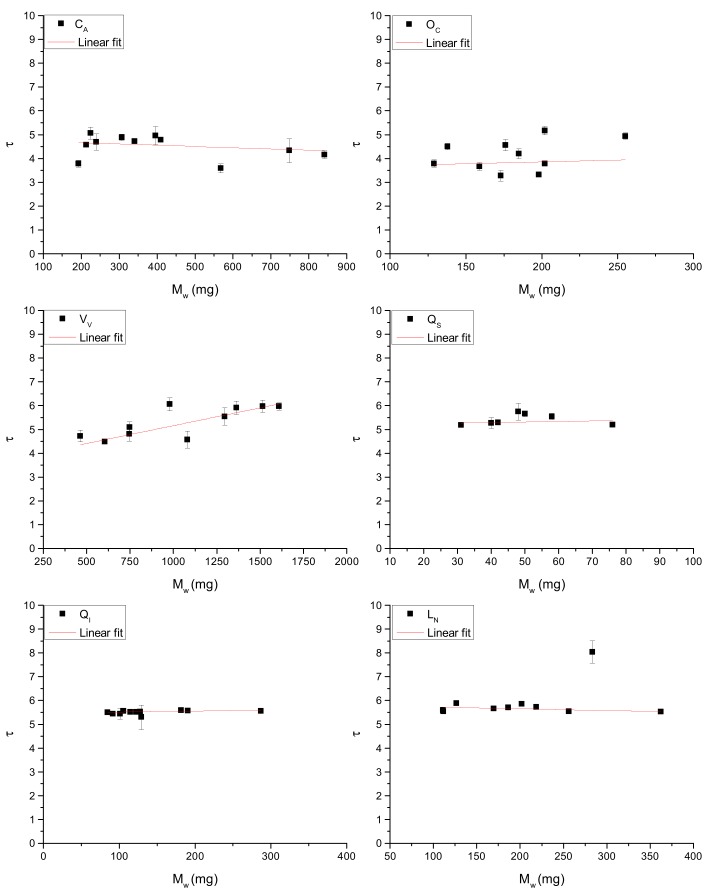
Relationships between *τ* and M_W_ in the investigated species.

**Figure 4:**
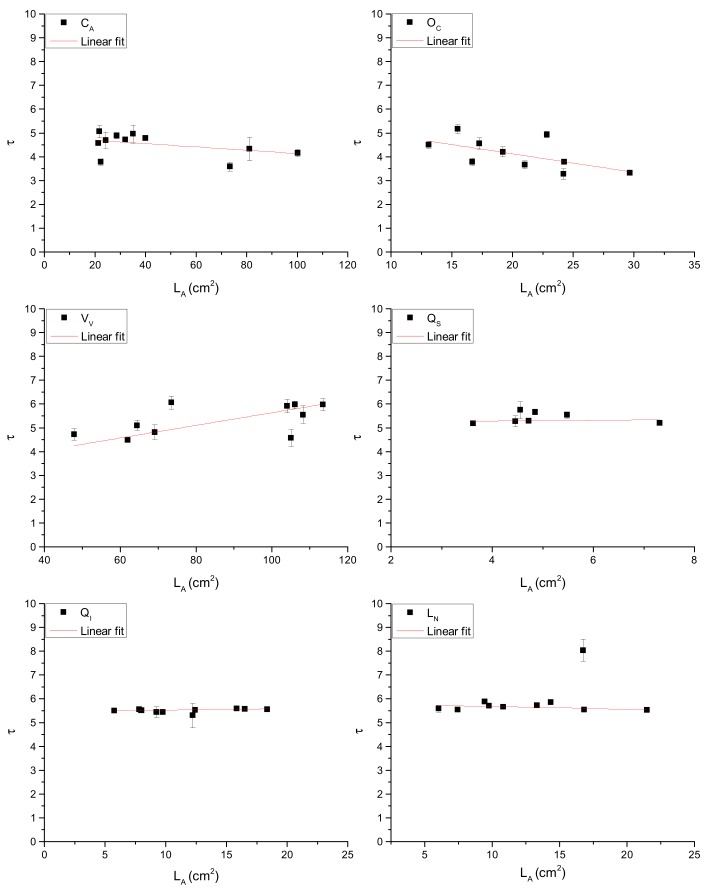
Relationships between *τ* and L_A_ with respect to the investigated species.

This study confirms the effectiveness of our method for measuring leaf water content using a THz-QCL based on leaf sampling and a linear equation. It relates the leaf water content to the product between the leaf optical depth and the projected area in a variety of different plant species. More specifically, our method can be used when non-invasive assessments are required. This method relies on the fact that the optical absorption of 2.55 THz radiation by a leaf is mainly due to water absorption; in fact, the water absorption coefficient in this spectral region can be close to 500 cm^−1^ [39], while scattering of long-wavelength radiation is generally considered negligible. As suggested by Baldacci et al. [28], if the optical depth (*τ*) is dominated by water absorption, *τ* may be expressed as:

*τ* = *αd_w_*, (2)

where α is the water absorption coefficient and *d_w_* is the effective water thickness inside the leaf. *d_w_* can be expressed as the ratio between water volume (hence water content, since water density is well known, *p_w_* = ~1000 mg cm^−3^) and the leaf area. In this way, Equation (2) can be simply rewritten as:

(3) $$\tau =K\frac{M_{w}}{L_{A}},$$

where K is the ratio between α and *p_w_*. Equation (3) helps us to understand why a simple correlation between *τ* and M_w_ or *τ* and L_A_ (Figure 3 and Figure 4) cannot be identified. 

This is better illustrated in Table 2, where a significant correlation between *τ* and M_w_ is only possible for *Vitis vinifera* L. and *Quercus ilex* L. For a better visualization of the experimental results, Equation (3) can be rewritten as:

(4) $$\tau L_{A}=K\;M_{w},\;$$

where K is now the slope coefficient of a linear relation [28]. A more classical treatment must take residual absorption and/or scattering into account by adding a C_0_ intercept coefficient. Therefore, the following linear relation holds:

*τ*L_A_ = C_1_M_w_ + C_0_. (5)

As shown in Table 2 and Figure 2, there is a high correlation between *τ*L_A_ and M_w_ for all of the species under study. The linear fit (red line) includes the following parameters: The intercept (C_0_), which is mainly attributed to residual absorption from the leaf dry mass, and the slope (C_1_), which is related to the absorption coefficient of water. The intercept C_0_ does not provide useful information on leaf water content and is equal to zero in most cases; therefore, its value was not reported. 

As highlighted by Toome [40], a decrease in SLA describing leaf morphology could be due to a thicker epicuticular wax layer. For this reason, we used species groups such as Q_I_ and L_N_ with lower SLA than the others. A significantly higher SLA in V_V_ suggests that these leaves were not affected by wax thickness. Although it was not possible to measure the thickness of the wax layer, results showed that irrespective of wax coating thickness, our THz measurements were able to provide absolute water content values for different leaves of different plants of all species investigated. 

Our method is not only useful for predicting M_w_ in many different kinds of species, but other interesting applications can be foreseen. For example, it could be possible to investigate the relative leaf water losses due to transpiration in real time. If interested in relative changes, no absolute calibration would be necessary. Regardless, the calibration method described in this study needs to be applied to just a limited number of leaves from a plant species with the successive detachment for determining leaf water content. Once this calibration is performed, further measurements on other leaves and/or plants can then be used to measure leaf water content nondestructively and monitor it over time even under field conditions. 

As highlighted by Winterhalter [41], canopy water content can be affected by various irrigation treatments and genetic profiles. The regulatory mechanisms controlling canopy water content are similar to those observed at leaf scale, although it is controlled by both vegetation and soil factors, such as soil moisture, atmospheric CO_2_ concentration, air temperature and humidity, solar radiation, and by physiological factors such as canopy conductance and leaf area index [42,43]. Water content is the result of the combined effects of processes such as photosynthesis, respiration, evaporation, and transpiration. It is therefore essential to determine the photosynthetic limitations in response to water stress by correlating photosynthetic rates and tissue water relations (e.g., leaf water potential or relative water content) in order to characterize the performance of crop species and improve water demand management, especially in the Mediterranean environment where water scarcity often occurs. Moreover, an invasive approach is required for measuring leaf water potential, which is one of the most common plant physiology parameters. With our nondestructive method, it is possible to estimate leaf water content without damaging the plant. This information could be used in conjunction with parameters or technologies that are more directly associated with leaf water potential. A useful technology in this respect is NIR (near-infrared reflectance), which is widely used for analyzing vegetable tissue [44]. In contrast to the THz region, however, in the near-infrared region, pigment absorption and scattering represent a relevant background that can hinder the water absorption information [45]. Although water content is not a measure of hydraulic status, it can be combined with nondestructive technologies to monitor leaf water content at both short- and medium-time scales. This may help plant physiologists to design and implement new experiments that can provide valuable insights into agricultural water management under variable environmental conditions. 

## 4. Conclusions

This study confirms the effectiveness of the method reported by Baldacci [28] for measuring leaf water content using a THz-QCL. The product *τ*L_A_ showed a close correlation with leaf water content for all of the species investigated at 2.55 THz. This nondestructive method can therefore be used for continuously monitoring leaf water content over time. By combining our technique with a gas exchange analyzer and physiological indices such as WUE (Water Use Efficiency) and/or FMC, experiments can be conducted to investigate plant physiological aspects both in the laboratory or in the field. Using our approach, it may be possible to investigate leaf water loss due to transpiration in real time. Furthermore, it is possible to examine the effects of environmental variations (e.g., variations in light quality) or other external stimuli on leaf water content. In the future, the THz-QCL approach might prove useful for estimating canopy water content and for indirectly monitoring the kinetics of leaf invasion by pathogenic bacteria, thus encouraging the development of specific models and countermeasures.

## 5. Patents

The method showed here has been the object of a patent application (no. 102,016,000,106,179). 

## Figures and Tables

**Table 1:** Leaf water mass (M_w_), leaf area (L_A_), leaf dry mass (LDM), and specific leaf area (SLA) of each species investigated (average ± SD). Data that do not share superscript letters differ significantly (Tukey’s multiple test) at *p* ≤ 0.05.

Parameters	C_A_	O_C_	Q_S_	V_V_	Q_I_	L_N_
Mw (mg)	407.18 ± 221.43 a	181.70 ± 36.18 a,b,d,e	49.29 ± 14.52 b,e	1040.40 ± 397.13 c	139.55 ± 59.22 d,b,c,e	202.75 ± 81.00 e,a
LA (cm^2^)	43.64 ± 27.82 a	20.38 ± 4.99 b,c,e,f	5.00 ± 1.16 c,e,f	85.35 ± 24.19 d	11.27 ± 4.14 e,f	12.62 ± 4.80 f
LDM (mg)	231.45 ± 106.95 a	118.50 ± 22.32 b,c	41.86 ± 10.82 c	263.70 ± 145.44 a	155.91 ± 63.69 a,b,c	193.25 ± 67.20 a,b
SLA (cm^2^ mg^−1^)	0.18 ± 0.04 a,b	0.17 ± 0.02 a,b	0.12 ± 0.01 b,d,e	0.38 ± 0.13 c	0.07 ± 0.02 d,e	0.06 ± 0.01 e

**Table 2:** Optical depth (*τ*; average ± SD) and the adjusted R^2^ values of the linear regression models of each species (ns = not significant; * = significantly different from zero at *p* ≤ 0.05).

	C_A_	O_C_	Q_S_	Q_I_	L_N_	V_V_
τ	4.50 ± 0.48	4.12 ± 0.66	5.42 ± 0.23	5.50 ± 0.08	5.91 ± 0.76	5.32 ± 0.64
τ vs. M_w_	−0.0526 ns	−0.1152 ns	−0.1352 ns	0.4176 *	0.1755 ns	0.8187 *
τ vs. L_A_	0.0582 ns	0.4569 *	−0.1802 ns	0.5918 *	0.1409 ns	0.6642 *
τ L_A_ vs. M_w_	0.9901 *	0.9052 *	0.9636 *	0.8637 *	0.9223 *	0.9837 *
